# Chronic cobalt exposure modifies nociception and orofacial inflammatory pain

**DOI:** 10.1186/1129-2377-14-S1-P87

**Published:** 2013-02-21

**Authors:** T Alexa, A Dondas, A Luca, VA Grigoras, OC Mungiu, CR Bohotin

**Affiliations:** 1 Centre for the Study and Therapy of Pain, University of Medicine and Pharmacy “Grigore T Popa” Iasi, Romania; 2 Department of Neurology, Rehabilitation Hospital, University of Medicine and Pharmacy “Grigore T Popa” Iasi, Romania; 3 Centre for the Study and Therapy of Pain, Department of Oncology, University of Medicine and Pharmacy “Grigore T Popa” Iasi, Romania

## Introduction

Cobalt chloride (CoCl2) is a widely used hypoxia-mimetic; preconditioning with cobalt has been reported to exert protection against ischemic injury as well as decreasing pro-inflammatory cytokine levels ( Shukla et al. 2011 ). Exposure to moderate hypoxia may lead to neurological phenomena such as paraesthesia, numbness or pain, possibly as a consequence of the modifications that appear in small DRG neurones. ( Gruss et al. 2006 ).

## Purpose

to investigate the effects of chronic (CoCl2) administration on nociception and orofacial formalin-induced pain in mice.

## Methods

Sixteen Swiss male mice were divided into 2 groups: control (n=8, i.p. saline) and chronic (CoCl2) (n=8, 12.5 mg/kg, i.p. 21 days). Tail flick (TF), hot plate (HP), mechanical (electronic von Frey aesthesiometer) and thermal (Hargreaves method) withdrawal thresholds were measured before cobalt/saline administration and thereafter every two days. 24 hours after the last dose, each group was evaluated for the nociceptive tests (TF&HP) and heat/mechanical hyperalgesia. Results were compared with the baseline using paired Student’s t test. After the nociceptive tests, the mice were injected with 20 ìl of 5% formalin into the upper right lip in order to assess the orofacial pain. The results were compared with saline group using unpaired Student’s t test.

## Results

Chronic (CoCl2) administration, significantly increased nociception in TF (p<0.001) and HP (p<0.001) tests and decreased the mechanical withdrawal threshold (p<0.02). On orofacial formalin pain, (CoCl2) tends to exert a pronociceptive effect on the first phase (p=0.09) and produces a significant antinociceptive effect (p=0.04) on the inflammatory phase.

## Conclusion

Taken together, our results demonstrate that chronic cobalt exposure increases acute nociception (TF, HP and first phase of the orofacial formalin pain) but has an antinociceptive effect on the inflammatory pain induced by orofacial formalin injection.
